# Mroh1, a lysosomal regulator localized by WASH-generated actin

**DOI:** 10.1242/jcs.197210

**Published:** 2017-05-15

**Authors:** Peter A. Thomason, Jason S. King, Robert H. Insall

**Affiliations:** Cancer Research UK Beatson Institute, Garscube Estate, Switchback Road, Glasgow G61 1BD, UK

**Keywords:** Mroh1, WASH, F-actin, HEAT repeats, *Dictyostelium*

## Abstract

The steps leading to constitutive exocytosis are poorly understood. In *Dictyostelium* WASH complex mutants, exocytosis is blocked, so cells that take up fluorescent dextran from the medium retain it and remain fluorescent. Here, we establish a FACS-based method to select cells that retain fluorescent dextran, allowing identification of mutants with disrupted exocytosis. Screening a pool of random mutants identified members of the WASH complex, as expected, and multiple mutants in the conserved HEAT-repeat-containing protein Mroh1. In *mroh1* mutants, endosomes develop normally until the stage where lysosomes neutralize to postlysosomes, but thereafter the WASH complex is recycled inefficiently, and subsequent exocytosis is substantially delayed. Mroh1 protein localizes to lysosomes in mammalian and *Dictyostelium* cells. In *Dictyostelium*, it accumulates on lysosomes as they mature and is removed, together with the WASH complex, shortly before the postlysosomes are exocytosed. WASH-generated F-actin is required for correct subcellular localization; in WASH complex mutants, and immediately after latrunculin treatment, Mroh1 relocalizes from the cytoplasm to small vesicles. Thus, Mroh1 is involved in a late and hitherto undefined actin-dependent step in exocytosis.

## Abstract (precis)

**Highlighted Article:** An unusual FACS-based screen using *Dictyostelium* identifies Mroh1 – a conserved protein with HEAT repeats – as a WASH and F-actin target that is important for constitutive exocytosis of lysosomes.

## INTRODUCTION

Membrane-bound compartments within eukaryotic cells have distinct structures and functions. Trafficking of contents between compartments, as occurs during endocytosis and biosynthetic secretion, relies on the targeting of small vesicles to specific locations. These vesicles carry membranous and soluble cargoes to their intended destinations, in a process that is highly regulated, and involves coordination of signalling and mechanical processes (Huotari and Helenius, 2011).

It has recently become appreciated that a major catalyst regulating structural transitions and formation of trafficking intermediates on endosomal and lysosomal compartments is branched polymerized actin (F-actin; Gautreau et al., 2014). This is generated in response to Arp2/3 activation by the WASH complex [Wiskott–Aldrich Syndrome protein homologue complex, consisting of the five members WASH (also known as WASHC1), Fam21 (WASHC2; encoded by three genes in mammals, *WASHC2A*, *WASHC2C* and *WASHC2D*), ccdc53 (WASHC3), SWIP (WASHC4) and Strumpellin (WASHC5); Derivery et al., 2009; Gomez and Billadeau, 2009; Jia et al., 2010]. WASH function is required for the structural integrity of endosomes and lysosomes (Derivery et al., 2012; Duleh and Welch, 2010; Gomez et al., 2012) and for the correct trafficking of a range of cargoes from endosomes to the plasma membrane (Derivery et al., 2009; Puthenveedu et al., 2010; Zech et al., 2011), the trans Golgi network (Gomez and Billadeau, 2009; Harbour et al., 2010) and lysosomes (Carnell et al., 2011; Duleh and Welch, 2010; Park et al., 2013; King et al., 2013). Recruitment of the WASH complex to endosomes is through binding of the Fam21 subunit to the Vps35 subunit of the retromer cargo-selective complex (Harbour et al., 2012; Helfer et al., 2013; Jia et al., 2012). In mammalian cells, the persistence of retromer on late endosomes as they mature and fuse with lysosomes may also account for the presence of WASH on lysosomes, as both complexes have been found on a portion of lysosome-associated membrane protein 1 (LAMP1)-positive membranes in mouse embryonic fibroblasts (MEFs) (Gomez et al., 2012). In *Dictyostelium*, although the WASH complex and retromer have been found on early endosomes and lysosomes, and appear to associate via the tail of the Fam21 subunit of the WASH complex, their recruitment does not appear to require one another (Buckley et al., 2016).

Disruption of the WASH complex halts the maturation and trafficking of lysosomes in *Dictyostelium* (Carnell et al., 2011; Park et al., 2013) and phagolysosomes in *Drosophila* macrophages (Nagel et al., 2017). In *Dictyostelium*, instead of undergoing their normal transition into neutral post-lysosomes from *wash*-knockout (KO) cells remain acidic and fail to undergo exocytosis (Carnell et al., 2011). This is due to the failure of the vacuolar/vesicular ATPase (V-ATPase) present in the lysosomal membrane (Marshansky and Futai, 2008) to recycle off the lysosome. In response to WASH delivery, F-actin causes the clustering of V-ATPase, which then segregates into small recycling vesicles that depart the lysosome (Carnell et al., 2011). Loss of WASH blocks both processes. As the V-ATPase has F-actin-binding domains (Holliday et al., 2000; Huss et al., 2011), there could be direct coupling between WASH-generated F-actin and the V-ATPase that coordinates its trafficking. Recent work has also found that WASH and V-ATPase can be co-immunoprecipitated from *Drosophila* cells, suggesting a possible direct interaction between the complexes (Nagel et al., 2017).

To investigate the regulation of the lysosomal trafficking pathway in more detail, we performed a screen based around the blocked exocytosis of WASH mutants. *Dictyostelium* is an excellent organism for analysis of the genetics of constitutive exocytosis, as exemplified by the recent demonstration of an exocytic function for mucolipin (Lima et al., 2012). We used a library of insertional mutants and selected those having disrupted exocytosis of fluorescent dextran. Among the mutants identified was one in the *mroh1* (also known as *heatr7a*) gene, encoding a large multiple HEAT-repeat-containing protein. We have characterized the cellular roles of Mroh1 and find that it strongly colocalizes on lysosomes with the WASH complex, and appears to be intimately involved with its cellular function.

## RESULTS

### Screening for mutants with disrupted exocytosis

**Fig. 1.**
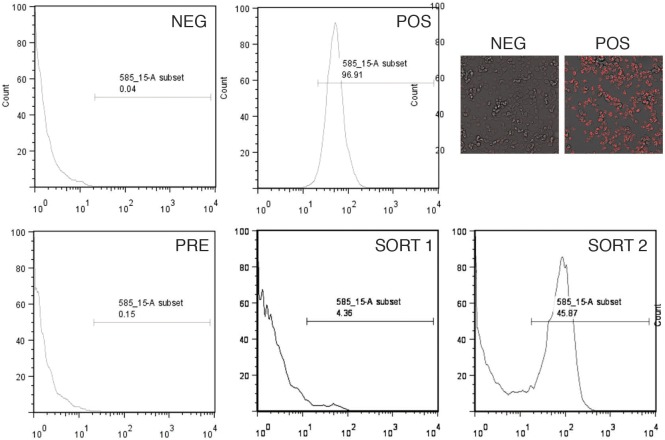
**Screen for exocytosis mutants.** Cells were labelled in TRITC–dextran overnight, then chased in fresh medium for 2–3 h. They were sorted by FACS, and the positive pool retained. WT cells were used to set the negative (NEG) window, and *wash*-mutant cells the positive (POS) window. The input cells from the REMI library indicate the presence of 0.1–0.2% positive cells prior to sorting (PRE), which is enriched to ∼5% after the first sort (SORT 1) and 50% after the second (SORT 2). Images of the negative and positive cells are also shown.

Our approach was based on the principle that *wash* mutants fail to efficiently exocytose indigestible material such as fluorescent dextran (Carnell et al., 2011). We generated a library of restriction enzyme-mediated insertion (REMI; Kuspa, 2006) mutants and screened for those with disrupted exocytosis. Pools of clones from the REMI library were labelled with tetramethylrhodamine isothiocyanate (TRITC)–dextran overnight, then allowed to exocytose in fresh medium for up to 3 h. Fluorescence-activated cell sorting (FACS) was used to select those cells that were still fluorescent after this time: wild-type (WT) cells exocytose all of their fluorescent dextran within ∼90 min, so those retaining signal at 3 h have a strong defect. Collected cells were expanded in culture and then put through a second round of FACS in the same manner. The proportion of positive cells in the initial input (library) was 0.05–0.2%. Positive cells became enriched to ∼5% of the total after the first sort and up to 50% after the second sort (Fig. 1). Cells were cloned (in 96-well plates) after the second sort.

Positive clones from the FACS selection were re-tested by labelling and microscopy to confirm their TRITC-retention phenotypes. Fig. 1 shows the typical appearance of mutant cells compared to normal cells in this assay.

### Identification of mutants

From the initial screen, 37 positive clones were taken forward, with gene identification for 15 clones being successful. One encoded Strumpellin, a member of the WASH complex. This finding confirmed that the library contained relevant mutants and that the screen was able to isolate and identify them.

Three of the other mutant clones all identified one protein, Maestro HEAT-like repeat-containing protein family member 1 (Mroh1). Indeed, these three mutants all had the same REMI insertion site in the genome, implying that the mutants (clones 2, 16 and 36 from the screen) were the same clone that had been amplified during library construction and/or the FACS procedure. The insertion site was identified as position 5048 of the *mroh1* genomic locus (numbered from the ATG translation start site), Dictybase accession number DDB_G0291161 (http://dictybase.org; Chisholm, 2006). The gene contains three small introns and has a total length of 5354 nucleotides (4934 coding), indicating that the REMI insertion site is close to the 3′ end.

### Mroh1 protein

The *Dictyostelium* *mroh1* gene encodes a large protein of 1647 amino acids with a predicted molecular mass of 186 kDa. As the name suggests, Mroh1 is predicted to contain HEAT repeats (HEAT stands for huntingtin, elongation factor 3, PR65 subunit of PP2A, and target of rapamycin; Andrade et al., 2001) similar to those found in the protein Maestro, a much smaller protein of unknown function (Smith et al., 2003). A typical HEAT repeat has two anti-parallel α-helices of ∼20 amino acids separated by a turn, and belongs to the Armadillo superfamily (Andrade et al., 2001). Secondary structural modelling of Mroh1 using a variety of tools, including Interpro (Mitchell et al., 2015; https://www.ebi.ac.uk/interpro/), DomPred (Buchan et al., 2013; http://bioinf.cs.ucl.ac.uk/psipred/), Phyre2 (Mezulis et al., 2015) and I-TASSER (Yang et al., 2015), all predict an entirely helical protein which, by virtue of its size, could contain ∼36 HEAT repeats (72 helices). The human Mroh1 orthologue (UniProt code Q8NDA8) has previously been predicted to contain seven HEAT-like repeats (PROSITE PRU00103) – and for this reason until recently the gene was known as HEATr7A – but this is almost certainly a substantial underestimate. By comparison, the XMAP215 family of microtubule polymerases contains 30 Maestro-like HEAT repeats (Fox et al., 2014) and the PR65/A subunit of protein phosphatase 2A (PP2A) has 15 tandem HEAT repeats (Groves et al., 1999).

Mroh1 orthologues exist throughout the eukaryotic kingdom, and the gene is very highly conserved both in size and in sequence. The *Dictyostelium* and human sequences are 27% identical and 50% similar, with the homology extending throughout their whole length. Table 1 shows the similarity of Mroh1 proteins across a selection of eukaryotes, using the *Dictyostelium* and human sequences as queries in both BLASTP (Altschul et al., 1990; http://ncbi.nlm.nih.gov/blast) and HMMER searches (Finn et al., 2015; http://ebi.ac.uk/Tools/HMMER). Multiple sequence alignment of these proteins (not shown) emphasizes the strength of the conservation of both sequence and size across a diverse range of organisms.

**Table 1.**
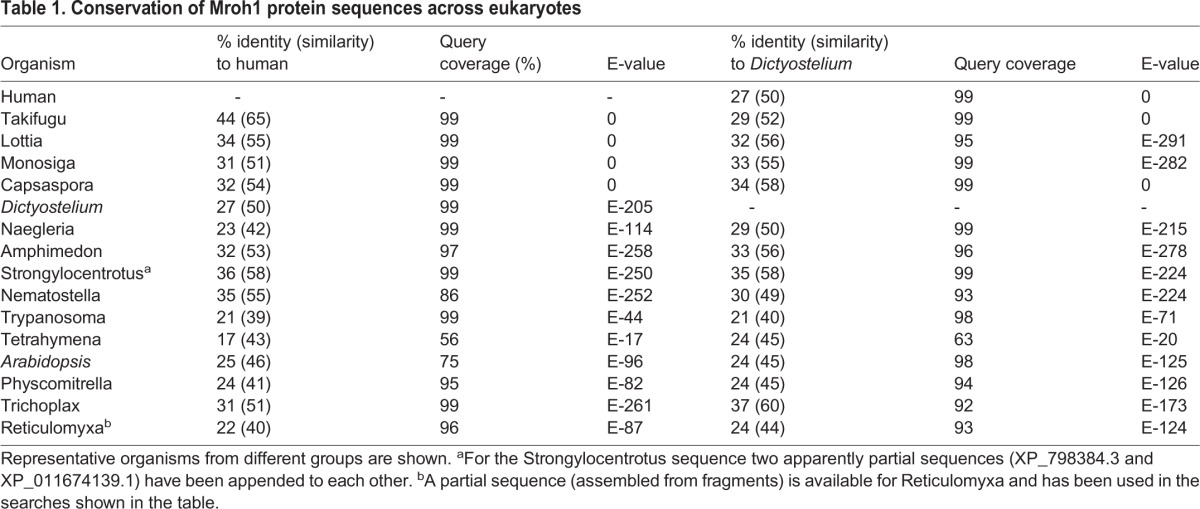
**Conservation of Mroh1 protein sequences across eukaryotes**

Mroh1 orthologues are distributed across most of the eukaryotic tree, with representatives in six of the seven recognized supergroups (phylogenomic classification according to Burki, 2014). It appears to be absent from Stramenopiles. For comparison, the WASH complex is more broadly conserved and can be found in all supergroups (Table S1). Within the Opisthokonts, Mroh1 is notably absent from fungi, which is also the case for the WASH complex (Derivery and Gautreau, 2010; Veltman and Insall, 2010).

### Confirmation of mutant and characterization of phenotype

To confirm that the REMI insertion into the *mroh1* gene was responsible for the observed phenotype we disrupted it by homologous recombination. We found that these clean genetic mutants had the same phenotype as the REMI mutant clones, and have used these new mutants throughout this study.

**Fig. 2.**
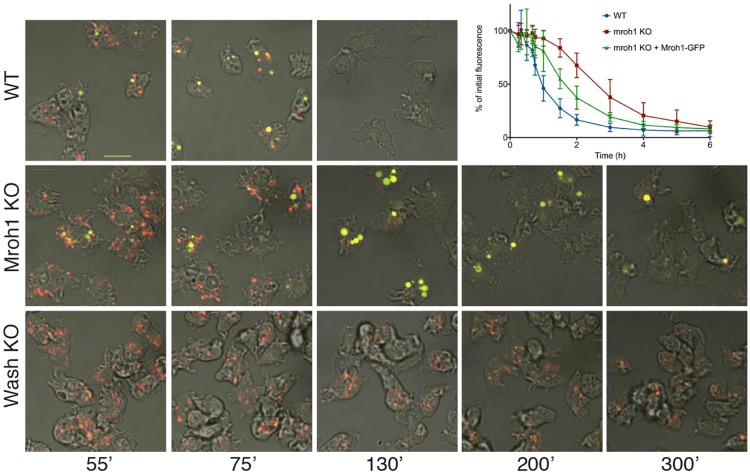
***mroh1* mutant has delayed exocytosis of neutral post-lysosomes.** Cells incubated with a mixture of TRITC– and FITC–dextran were imaged by confocal microscopy at the times shown (after addition of the fluorescent dextran). Acidic vesicles are red, whereas vesicles that have neutralized are yellow. Lysosomes in *mroh1*-KO cells neutralize at the same time as those in WT cells, but exocytosis is significantly delayed. In contrast, lysosomes in *wash*-KO cells remain acidic. To quantify the rate of exocytosis in WT, *mroh1*-KO and rescue cells, cells that had been labelled to equilibrium with TRITC–dextran were allowed to exocytose in fresh medium, and their remaining fluorescence was measured at the indicated times (graph). Results show the mean±s.d. The time taken to reach 50% of initial fluorescence for each strain (mean±s.d., in minutes) was 60.2±8.5 for WT, 160.2±33.4 for KO, and 98.7±21.0 for rescue. For each replicate (11 for WT, 18 for KO, 7 for rescue) the area under the curve was calculated then statistical analysis was performed by ANOVA followed by Tukey's HSD test. This showed that each pair of cell types was significantly different (WT versus KO *P*-val=0; KO versus Rescue *P*-val=0.00026; WT versus Rescue *P*-val=0.003), confirming that Mroh1–GFP provides a partial rescue. Scale bar: 10 μm.

The *mroh1* mutant has disrupted exocytosis compared to WT cells. Unlike the *wash* mutant, the phenotype is not one of complete blockage, but rather a delay of exocytosis (Fig. 2). The half-time of exocytosis in WT cells is ∼60 min, compared to 160 min in *mroh1* mutant cells.

Using a mixture of pH-insensitive (TRITC) and pH-sensitive (fluorescein isothiocyanate, FITC) dextran, we observed that the characteristic defect in *mroh1* mutants was the presence of prominent neutral vesicles (usually between one and three per cell). Rather than being rapidly exocytosed as in WT cells, these post-lysosomes persisted in the cell for two or more hours (Fig. 2). Expression of a C-terminally GFP-tagged version of Mroh1 in the mutant significantly restored exocytosis (Fig. 2) confirming that the disruption of Mroh1 was responsible for the observed phenotype. As assessed with microscopy (see below), it was clear that not every cell expressed visible Mroh1–GFP, and in those that did the fluorescence intensity varied considerably. Therefore, it is probably not surprising that the Mroh1–GFP construct did not provide a complete rescue of the *mroh1*-KO phenotype. The ability of the *mroh1* mutant cells to neutralize their lysosomes suggests that Mroh1 functions at a later step than the WASH complex (see below).

### Mroh1 accumulates around lysosomes

**Fig. 3.**
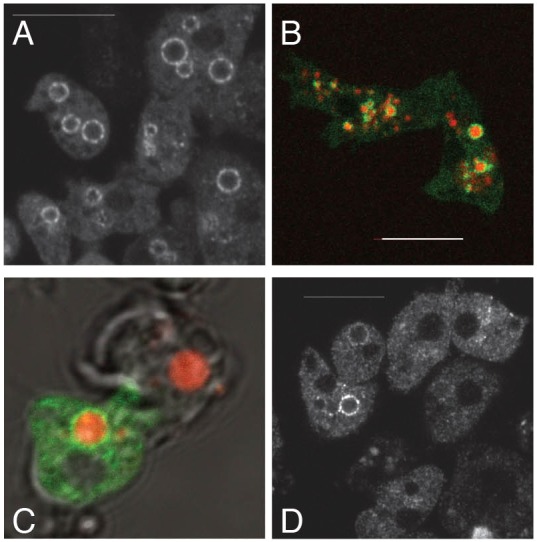
**Mroh1 localizes to lysosomes.** (A) Mroh1–GFP expressed in cells localizes to lysosomes. (B) When fluorescent dextran is included in the medium, this is endocytosed and transported into these same vesicles. (C) The mouse Mroh1 gene cloned into a *Dictyostelium* C-terminal GFP vector also localizes to these same compartments. (D) When the first 14 codons of Mm_MROH1 are optimized for expression in *Dictyostelium*, the expression level is higher and the localization of Mm_MROH1 to lysosomes clearer. Scale bar: 10 μm.

As Mroh1 was identified from a screen for exocytic mutants, we tested whether it localized to a relevant compartment in the cell. Mroh1–GFP was clearly enriched on several intracellular vesicles per cell (Fig. 3A). The Mroh1–GFP presents a patchy localization on these vesicles, rather than a continuous coat, in a manner that is very reminiscent of the localization of the WASH complex. When fluorescent dextran was added to the medium, these vesicles also became labelled in their lumens (Fig. 3B), indicating that they are derived from endocytic compartments.

The conservation of Mroh1 led us to test the expression of a mammalian Mroh1 orthologue fused to GFP in *Dictyostelium*, and see whether it had the same localization as the native protein. We expressed the mouse Mroh1 cDNA fused to a C-terminal GFP tag (Mm-Mroh1–GFP) in *Dictyostelium* cells. The mouse gene is an approximately equal size to the *Dictyostelium* gene but has a very different codon usage. We found that it was expressed at a very low level in *Dictyostelium* (0.1% of that of Dd-Mroh1–GFP as determined by western blotting; not shown). In those few cells that expressed detectable levels of Mm-Mroh1–GFP, it was clearly localized to dextran-containing lysosomes like the native protein (Fig. 3C). To improve the expression of Mm-Mroh1–GFP we examined the 5′ end of the coding sequence for rare codons, and found that 12 of the first 14 codons were potentially problematic. With these codons optimized, the GFP-tagged Mm-Mroh1 cDNA expressed ∼10-fold better, and again localized very clearly to lysosomes (Fig. 3D). However, neither the original mouse cDNA nor the altered version was able to rescue the exocytosis defect of the *mroh1*-KO mutant, presumably because the expression was too low.

As the vesicles identified by Mroh1–GFP have an endocytic origin, we wished to understand at what stage during the endocytic trafficking pathway Mroh1 started to accumulate on them. We imaged cells expressing Mroh1–GFP that had been pulse-labelled with TRITC–dextran. By performing time-lapse imaging of these dextran-filled vesicles, we found that Mroh1–GFP was first enriched on occasional vesicles at 25–30 min after endocytosis, but it took ∼50 min before a large number of vesicles showed Mroh1 accumulation (Movie 1). This is similar to the time of appearance of neutral post-lysosomes in the cell, and suggests that Mroh1 builds up late in the lysosomal pathway.

To test in greater detail for evidence of an earlier phase of recruitment of Mroh1–GFP onto endocytic vesicles we co-expressed this protein with the mCherry-tagged PH domain of CRAC [a phosphatidylinositol (3,4,5)-trisphosphate (PIP_3_)-binding protein that localizes to newly forming macropinosomes; Veltman et al., 2014, 2016]. We performed rapid time-lapse imaging of the formation of macropinosomes. We were able to clearly image their uptake, and thus observe the formation of new endosomes (Movie 2). Although strongly labelled on their surface with PH-CRAC, these early endosomes did not show presence of Mroh1–GFP. Thus, it appears that Mroh1 is not recruited to early endosomal vesicles but only onto mature lysosomes.

### Colocalization of Mroh1 with the WASH complex

**Fig. 4.**
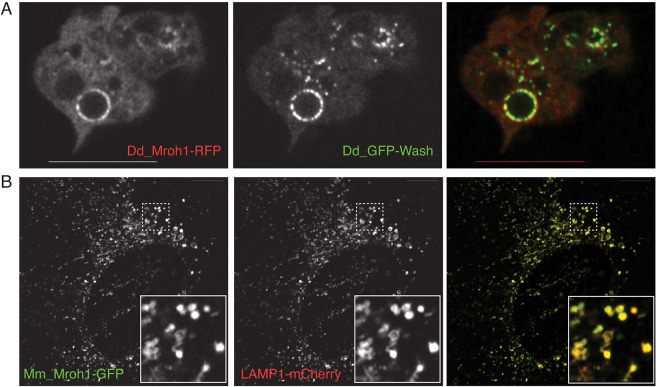
**Colocalization of Mroh1 and the WASH complex.** (A) Confocal section of wild-type *Dictyostelium* cells co-expressing Dd-Mroh1–RFP and GFP–WASH, showing colocalization to circular lysosomes. (B) Confocal section of MEFs co-expressing Mm-Mroh1–GFP+LAMP1-mCherry, showing colocalization to lysosomes dispersed throughout the cell. Insets in B are boxed areas at a magnification of ×3.2. All images are from Airyscan confocal using sequential channel capture. Scale bars: 10 μm.

The similarity in the appearance of Mroh1 on the surface of lysosomes to the known localization of the WASH complex (Park et al., 2013) led us to investigate their possible colocalization. Co-expression of Mroh1 with WASH (Fig. 4A; Movie 3) showed that they indeed strongly colocalize. This colocalization is not merely to the same vesicles, but to the same regions of these vesicles. To assess their colocalization more quantitatively, we performed radial analysis of the signals for each protein: the circumference of the vesicles was divided into 24 sections of 15° each, and measured for the intensity of the red and green fluorescence channels. These radial intensity profiles (Fig. S1) confirm two important features: (1) they are irregularly shaped, demonstrating the patchiness of the distribution of the proteins (a continuous coat would yield an approximately circular profile), and (2) they are very similar, commonly showing the same peaks and troughs, irrespective of their absolute signals. Thus, Mroh1 and the WASH complex localize to the same positions on the circumference of vesicles.

Interestingly, while there is no difference in the radial distribution of Mroh1 and the WASH complex, we were able to detect a displacement in their positions relative to the centre of the vesicle: Mroh1 intensity consistently peaked slightly to the outside of the WASH complex peak (i.e. more peripheral on the vesicle) (Fig. S2). When the diameter of vesicles was measured according to the peak-to-peak intensity of Mroh1 and the WASH complex, the measured size was, on average, 2.7 pixels greater for Mroh1 than for the WASH complex. This suggests an overall displacement in their positions across the vesicle of the order of 125 nm (∼60 nm each side). Although we used live-cell super-resolution imaging (Airyscan confocal), the resolution remains insufficient to give a precise value of the difference, but the data suggest that Mroh1 sits outside WASH on the cytoplasmic face of lysosomes.

To establish that Mroh1 is a genuine lysosomal protein we co-expressed Mm-Mroh1–GFP with mCherry-tagged LAMP1 (Xu and Ren, 2015) in MEFs. The proteins show a very high degree of colocalization (Pearson's Rr value 0.92±0.08, mean±s.d. from 11 images; Fiji CoLoc2 plugin; Fig. 4B), both of them extensively labelling the dispersed lysosomal network in these cells. These observations conclusively place Mroh1 on the lysosome in both *Dictyostelium* and mouse cells, and hence provide a new marker for this compartment in both systems.

Our exocytosis screen thus identified a conserved protein that both performs a cellular function related to that of WASH in regulation of vesicle traffic late in the endocytic pathway, and whose cell biology and localization within the same compartment are similar.

### Mroh1 and the WASH complex have different dynamic behaviour

We explored the dynamics of Mroh1 and WASH colocalization on vesicles by performing fluorescence recovery after photobleaching (FRAP) experiments. Cells expressing Mroh1–GFP or GFP–WASH were incubated in low fluorescence medium containing dextran (to enlarge vesicles) and then squashed lightly under a thin layer of agarose, in order to help keep vesicles in focus during imaging. Entire vesicles were bleached with a 405 nm laser, and the recovery of GFP signal monitored over time. Mroh1 and WASH had clearly distinguishable properties: Mroh1 fluorescence had a recovery half-time (geometric mean calculated from natural-log transformed data) of 2.70 s (95% confidence intervals 2.16, 3.36 s, *n*=32) compared to 4.78 s (95% confidence intervals 4.40, 5.16 s, *n*=23, *P*=0.015) for WASH (Movies 4 and 5). More clearly, and compounding the difference in half-time of recovery, WASH exhibited a much higher immobile fraction (74.9±14.0%, *n*=23) than Mroh1 (44.5±12.2%, *n*=32; Welch two sample *t*-test, *P*=1.4×10^−10^), meaning that WASH recovered to a much lower extent than Mroh1 after bleaching. Mroh1 therefore has a much more dynamic equilibrium on the vesicle surface, and more readily exchanges with a soluble pool of protein than does WASH. These findings are also consistent with our observations of Mroh1 assuming a more peripheral location on these vesicles, and indicate that it is less avidly bound to its target sites than is the WASH complex.

### Mroh1 does not stably associate with the WASH complex

The difference in the dynamic behaviour of Mroh1 and the WASH complex hinted that – despite their very particular colocalization on vesicles – they have different roles in the cell. To determine whether they are part of a stable complex, we co-immunoprecipitated Mroh1–GFP with the WASH complex by using GFP-trap beads (Trinkle-Mulcahy et al., 2008), followed by western blotting for endogenous WASH using an anti-WASH antibody (Park et al., 2013). GFP–Fam21 was used as a positive control, and easily co-precipitated WASH protein from cells (Fig. S3). In contrast, no WASH protein was co-precipitated with Mroh1–GFP. These results demonstrate that there is not a direct physical interaction between Mroh1 and the WASH complex, that any putative association does not occur among the soluble pool of the proteins when extracted from the cell or that any such association is not stable enough to be maintained in cell extracts.

### WASH complex localization and activity do not require Mroh1

Mroh1 and the WASH complex colocalize on vesicles but appear to have different properties and seem not to coexist in a stable multi-protein complex. We therefore tested whether they show independence, or some degree of mutual dependence, in their localization. We found that normal Mroh1 localization to vesicles required the WASH complex, but WASH complex localization was independent of Mroh1.

**Fig. 5.**
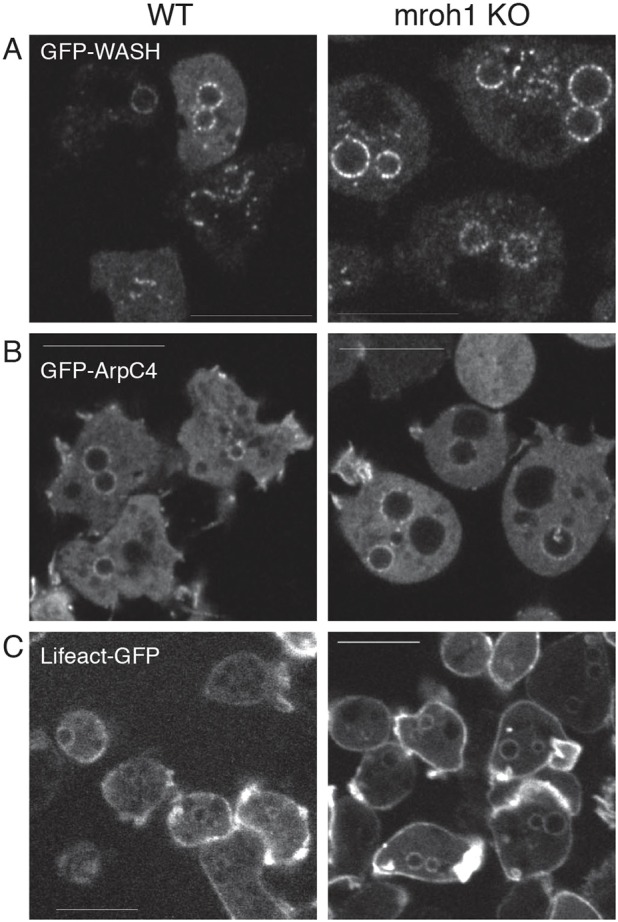
**The WASH complex localizes normally and is active in the *mroh1* mutant.** (A) GFP–WASH is correctly localized in *mroh1*-KO cells, although due to the *mroh1* mutation the vesicles to which it localizes are larger than those in WT cells. The signal on the vesicles was calculated and compared to the cytosolic signal, and found to be the same for both strains (2.77±0.21 for WT, 3.05±0.12 for *mroh1* KO, mean±s.d. *n*=29 for WT, *n*=63 for *mroh1* KO, *P*=0.24, Student's *t*-test). (B) Localization of GFP–ArpC4 to lysosomes and post-lysosomes in WT and *mroh1* KO cells. The vesicle-to-cytosol signal for WT cells was 1.47±0.02, and for *mroh1* KO cells was 1.49±0.02, mean±s.d. *n*>70 cells, *P*=0.51 (Student's *t*-test). (C) Localization of the Lifeact–GFP marker for F-actin in WT and *mroh1*-KO cells. Scale bars: 10 μm.

In *mroh1* mutant cells the localization of the WASH complex was not disturbed (Fig. 5A): it still localized to the lysosomes and post-lysosomes, and distributed in an equally patchy way. Therefore, WASH complex recruitment and distribution on lysosomes does not require Mroh1. It was apparent that the persistent post-lysosomes in *mroh1* mutant cells – labelled with GFP–WASH on their surface – were larger than those in WT cells. To assess the difference quantitatively, we measured the diameter of the vesicles and found them to be, on average, 27% larger in *mroh1* mutant cells than those in WT. Under these experimental conditions, the cross-sectional area of WT vesicles was 3.4±1.0 μm^2^ (mean±s.d.; *n*=32) versus 5.5±1.8 μm^2^ (*n*=44; Student's *t*-test *P*=2×10^−8^) for *mroh1* mutant cells. This equates to a 60% greater vesicle surface area and a doubling of volume (assuming a spherical vesicle).

To assess whether the WASH complex on these vesicles was functional, we transfected cells with reporters for the Arp2/3 complex (GFP–ArpC4; Fig. 5B) and F-actin (Lifeact-GFP; Fig. 5C). Both markers clearly localized to lysosomal vesicles in mutants. This demonstrates that the WASH complex does not require Mroh1 for normal Arp2/3 recruitment and actin polymerization. We did not observe any idiosyncratic fountain-like actin polymerization on *mroh1* mutant vesicles, which distinguishes them from *fam21* mutant cells, which like *mroh1*-KO cells also have enlarged neutral post-lysosomes (Park et al., 2013).

In WT cells, one direct consequence of WASH complex arrival onto lysosomes is the clustering and subsequent removal of V-ATPase on recycling vesicles, which induces neutralization of the lysosome to become a post-lysosome. This process requires the F-actin formed through the action of the WASH complex, although the mechanism of coupling is not yet understood. Because the arrival of WASH at lysosomes catalyses the rapid removal of V-ATPase, the two proteins are only transiently present on the same vesicle. Consistent with the fact that *mroh1* mutant cells do contain neutral post-lysosomes, we observed that the reciprocal localization of WASH and V-ATPase was preserved in these cells (Movie 6), further suggesting that WASH function is not impaired in the absence of Mroh1.

We also determined whether Mroh1 and V-ATPase showed a similarly reciprocal localization in WT cells. Co-expression experiments (Movie 7) demonstrate that vesicles that are strongly labelled with Mroh1 contain no V-ATPase and vice versa. We also observed vesicles that contain abundant V-ATPase and that also show the presence of one or two small dots of Mroh1 – just as is the case for V-ATPase and WASH.

### Mroh1 localization requires WASH-induced F-actin

**Fig. 6.**
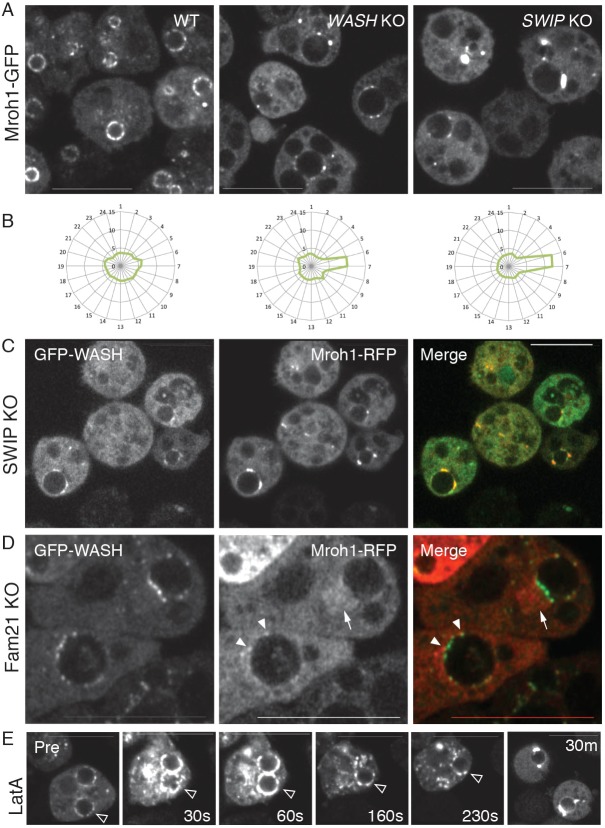
**Mroh1 localization requires the WASH complex.** (A) In *wash*- and *swip*-KO cells the distribution of Mroh1–GFP on lysosomes is disturbed, tending to form bright foci rather than a patchy coat. This is quantified in B, where 10 cells of each strain were analysed for their Mroh1–GFP distribution, this being shown as radial profile plots of the mean values (vesicles were oriented such that any foci were located at the right-hand side). (C) Mroh1–RFP and GFP–WASH colocalize in *swip*-KO cells, with most of the vesicular signal concentrated into puncta. (D) In *fam21*-KO cells some Mroh1–RFP is found on the enlarged post-lysosome (arrowheads) localizing adjacent to the GFP–WASH, or is seen in filaments that emanate from the post-lysosome (arrow). These filaments are thought to represent Mroh1 being removed from the post-lysosome under the influence of fountains of F-actin (see Movie 8). (E) Mroh1–GFP in cells treated with Latrunculin A resembles that in *wash*- and *swip*-KO cells. Panels show a Mroh1–GFP-labelled lysosome (open arrowhead) before and during exposure to 5 µM LatA. An immediate condensation of Mroh1–GFP onto vesicles is followed by a slower resolving of the Mroh1–GFP into characteristic foci on the surface of lysosomes. The cell in the final panel (without an arrowhead) is from a different experiment that had a 30-min exposure to LatA to show the later behaviour. Scale bars: 10 μm.

In *wash* and *swip* (a core member of the WASH complex) mutant cells, Mroh1–GFP was still able to localize to lysosomes, but its distribution was significantly altered compared to WT cells. In both mutants, instead of displaying a broad patchy distribution, the Mroh1–GFP tended to accumulate in one or a few concentrated foci on the lysosomal surface (Fig. 6A). This altered localization was confirmed by examining the radial intensity profile of the Mroh1–GFP (Fig. 6B) – in normal cells the distribution is spread unevenly all around the lysosomal perimeter, whereas in both *wash* and *swip* mutant cells the Mroh1–GFP is highly polarized, with the average distribution favouring a single aggregate at one point in the perimeter of each lysosome.

The appearance of Mroh1–GFP in *swip* mutants reminded us of the behaviour of WASH in these cells, which is non-functional and similarly delocalized (Park et al., 2013). We therefore co-expressed Mroh1–RFP with GFP–WASH in *swip* mutants: both proteins showed cytosolic and vesicular localization. Importantly, the co-expressed Mroh1 and inactive WASH that were present on these lysosomes colocalized in aberrant foci instead of in broad patches as in WT cells. Thus, loss of lysosomal F-actin causes the coalescence of WASH and Mroh1 into large patches. These findings for *Dictyostelium* lysosomes are reminiscent of those made for endosomes and lysosomes in mammalian cells in which vesicular F-actin has been inhibited; in those cells the compartments collapse and the WASH coalesces into large patches (Derivery et al., 2012; Duleh and Welch, 2010; Gomez et al., 2012).

We could also test what occurred in the opposite situation, excessive WASH activity and actin polymerization, by taking advantage of the unique behaviour of the *Dictyostelium fam21* mutant (Park et al., 2013). In these cells, there was less Mroh1 localized to the membrane of the enlarged post-lysosome, and close inspection showed it to be present in small puncta and diffuse regions near the surface. Co-expression of GFP–WASH with Mroh1–RFP in *fam21*-KO cells (Fig. 6D) revealed that the Mroh1 was located adjacent to the concentrated spots of WASH present on the post-lysosome surface. The unregulated WASH in *fam21* KO cells generates streams of F-actin flowing from the post-lysosome into the cytoplasm (Park et al., 2013). We observed Mroh1 associating with these actin streams (Fig. 6C; Movie 8). The disruption to Mroh1 localization in both WASH-null and WASH-hyperactive cells indicates that F-actin regulates the correct distribution of Mroh1 on lysosomes.

### Mroh1 redistribution by dynamic actin polymerization

These results led us to examine whether the proper distribution of Mroh1 on lysosomes specifically requires the normal presence of vesicular F-actin. WT cells expressing Mroh1–GFP were imaged by confocal microscopy while actin polymerization was blocked with Latrunculin A (LatA). The consequences are rapid and striking, but complex (Fig. 6E; Movie 9). Immediately upon LatA addition, the Mroh1 condenses onto cytosolic vesicles, including the lysosome. This acute response is followed by a prolonged second phase (taking up to 30 min) where most of the F-actin on small cytosolic vesicles is gradually lost, while the signal on the large lysosomes begins to resolve into one or two very intense foci.

There is a fundamental difference between the normal distribution of Mroh1 and the localization after prolonged LatA treatment. In untreated cells, the Mroh1 is present in small puncta that decorate the lysosomal surface, forming a ring of discrete spots (when seen in cross section). Chronic LatA treatment causes the accumulation of Mroh1 in large intense foci with no obvious punctate sub-structure. Importantly, this is the same distribution as seen in *wash* and *swip* mutants, again indicating that WASH-nucleated actin maintains the normal distribution decorating the lysosomes.

We conclude that initial Mroh1 recruitment to internal membranes and vesicles is not F-actin dependent, but its intracellular mobility and relocalization between vesicles completely requires F-actin. Since WASH and Mroh1 do not appear to associate directly with one another, this implies that WASH-catalysed actin polymerization drives Mroh1 off of vesicles to new locations within the cell.

### Behaviour of Mroh1 and WASH during exocytosis

The WASH complex is removed from post-lysosomes shortly before their exocytosis (Carnell et al., 2011). As Mroh1 was identified from an exocytosis screen and is present on the same vesicles as the WASH complex, we examined the dynamics of Mroh1 and WASH by performing time-lapse confocal imaging of vesicles being exocytosed.

**Fig. 7.**
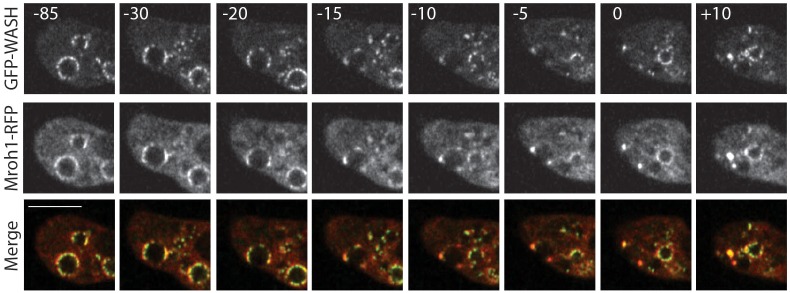
**Behaviour of Mroh1 and WASH during exocytosis.** WT cells co-expressing GFP–WASH (top row) and Mroh1–RFP (middle row) were imaged during vesicle exocytosis. The merged images are in the bottom row. The numbers indicate the time (in seconds) relative to exocytosis. The protein that remains on the vesicle transitions from a patchy to punctate pattern, and the two proteins remain colocalized. The protein foci remain on the plasma membrane for a brief period after the vesicle has been exocytosed. Scale bar: 5 μm.

Mroh1 and the WASH complex displayed identical behaviour during exocytosis (Fig. 7; Movie 10). Shortly before vesicle exocytosis, the signal intensity of the proteins on the vesicle surface was reduced, as some of the protein was removed. The protein that remained underwent a rapid transition in its distribution, from its usual patchiness to being highly concentrated into two or three foci. There followed a delay (varying between 10 and 60 s) between this transition and the exocytosis of the vesicle. As the vesicle was exocytosed, Mroh1 and the WASH complex remained completely colocalized, and upon exocytosis they were seen briefly on the cell surface as two or three large puncta that endured for less than a minute.

For quantitative analysis of this behaviour, we again traced the radial intensity profiles of the proteins on the vesicle circumference, this time tracking them prior to and during exocytosis. These results (Movie 11) confirm that Mroh1 and the WASH complex remained colocalized throughout this process.

Intriguingly, we were also able to image vesicle exocytosis in *mroh1* mutant cells: although exocytosis is greatly delayed in these cells, it is not completely blocked. The behaviour of the WASH complex at exocytosis is apparently normal in *mroh1* mutant cells: GFP–WASH partly departs the vesicle, and the remainder undergoes a patchy-to-punctate transition shortly prior to exocytosis, remaining on the plasma membrane for a short while thereafter (Fig. S4; Movie 12). Thus, both the aggregation of the WASH complex into large puncta and the fusing of post-lysosomes with the plasma membrane are able to proceed in the absence of Mroh1.

Finally, we tested how the departure of Mroh1 and WASH from vesicles immediately before exocytosis relates to other late exocytic events. Specifically, we looked at two markers of special interest: F-actin (using Lifeact-RFP) and the Arp2/3 complex (using RFP–ArpC4). Both markers are present on post-lysosomes prior to exocytosis, together with Mroh1 and WASH. Intriguingly, the peak intensity of Arp2/3 and F-actin signals, as shown by these markers, was seen immediately at the point of exocytosis during the expulsion of the vesicle from the cell (Movie 13 and 14). This occurred after most of the Mroh1–GFP (and therefore, by analogy, WASH) had resolved into foci and been removed from the surface of the vesicle, suggesting that this final phase of Arp2/3 and F-actin activity is mediated by another nucleator.

## DISCUSSION

We have developed a screen for mutants that increase our understanding of WASH function in the cell. In this paper, we report the isolation of a *mroh1* mutant that shows many similarities to the phenotypes of WASH complex mutants, and whose gene product intimately colocalizes with the WASH complex.

Despite the colocalization of Mroh1 and the WASH complex, Mroh1 is not required for WASH complex function: indeed, in *mroh1* mutants the WASH complex is still active, as indicated both by the neutralization of lysosomes into post-lysosomes at the normal time expected in WT cells and, more directly, by the presence of F-actin on post-lysosomes (F-actin being completely absent from lysosomes in *wash* mutant cells; Carnell et al., 2011). In WT cells, neutral post-lysosomes are rapidly exocytosed (Park et al., 2013), but in *mroh1* mutant cells they persist for ∼2 h before exocytosis, and also become substantially larger than those of WT cells. Consequently, these post-lysosomes sequester a higher proportion of the cellular WASH complex on their surface than do post-lysosomes in WT cells, which inevitably must have consequences for the normal vesicular trafficking cycles in *mroh1* mutant cells.

The molecular function of Mroh1 is not known. It belongs to a family of large HEAT-repeat-containing proteins whose roles include structural scaffolding and enzymatic regulation. Importin-β2 is a HEAT repeat protein that provides a binding scaffold for the small GTPase Ran (Vetter et al., 1999). The HEAT repeats of mTOR form distinct structural elements in the mTORC1 complex that scaffold the catalytic core and mediate dimer formation (Yang et al., 2016). PR65/A supports the binding of both the catalytic and regulatory subunits of PP2A (Shi, 2009) and may act as a flexible tension sensor to transduce mechanical inputs that regulate PP2A catalytic activity (Grinthal et al., 2010). One possibility for a function of Mroh1 could be to act as a sensor of vesicle size or membrane curvature, perhaps transducing this signal to an effector that controls subsequent vesicle trafficking steps.

Surprisingly, Mroh1 has so far escaped attention, despite the fact that it is a large gene that is extremely highly conserved across most eukaryotic supergroups, and thus easily recognized. Very little is known about the role of Mroh1 in cells. Only one functional report has been published, from a study of shoot gravitropism mutants in Arabidopsis. Here, Mroh1 (known as SHOOT GRAVITROPISM6, SGR6; Hashiguchi et al., 2014) is localized to the vacuole membrane (VM) in shoot endodermal cells, and is required for the normal gravitropic response mediated by the sedimentation of plastids called amyloplasts. These are dynamic compartments that are formed from invaginations of the vacuole membrane and which are also intimately associated with actin filaments. SGR6 may help to regulate the flexibility of the vacuole membrane so that the required invaginations can form properly. Just as with Mroh1–GFP expressed in *Dictyostelium*, GFP–SGR6 in plants showed a patchy, rather than smooth, localization to the vacuole membrane. It seems likely that the vacuolar membrane of these cells could have functional similarities to the vesicular/lysosomal membrane of *Dictyostelium* and other cells (de Marcos Lousa and Denecke, 2016) to which Mroh1 localizes.

A role for Mroh1 in normal membrane dynamics/vesicular transport is also supported by a screen carried out in HeLa cells for genes involved in the biosynthesis and delivery of the plasma membrane protein tsO45G (Simpson et al., 2012). By using an RNAi screening approach that individually targeted more than 3600 genes, the *mroh1* knockdown was identified as being among the top 2% of strongest inhibitors of plasma membrane trafficking of this protein. Although no mechanistic studies were performed, we envisage that impaired recycling of vesicles from the endolysosomal pathway in the *mroh1*-knockdown cells could have knock-on consequences for secretory vesicle trafficking. Unfortunately none of the WASH complex members were included in this screen and so a comparison with the effects of Mroh1 is not possible.

Mroh1 may be recruited to late endosomal/lysosomal membranes via the small GTP-binding protein Rab7. In a recent proteomic study from *Drosophila* cells, a HEAT repeat protein identified only as CG12132 was found to be a binding partner of Rab7 *in vitro*, and also to partially colocalize with it when they were co-expressed in S2 cells (Gillingham et al., 2014). Homology searches identify CG12132 as an Mroh1 orthologue, and the connection with Rab7 is consistent with the cellular localization of Mroh1 in both *Dictyostelium* and mammalian cells that we have shown here.

Recruitment of Mroh1 to lysosomes could therefore be achieved by Rab7 (just as recruitment of the WASH complex is ultimately a Rab7-dependent event, via the binding of retromer to Rab7), but the recruitment does not require F-actin. In contrast, the proper distribution of Mroh1 on lysosomes is dependent on F-actin, as is the case for WASH in *Dictyostelium* and mammalian cells. The collapse of endosomes and lysosomes that is caused in mammalian cells by the loss of vesicular F-actin is accompanied by the accumulation and coalescence of WASH into large aggregates on their surface (Derivery et al., 2012; Duleh and Welch, 2010; Gomez et al., 2012). This is very similar to our observations for Mroh1. Vesicular actin filaments therefore help to distribute Mroh1 and WASH around the surface, to restrict the accumulation of the proteins on these compartments, and – at least in the case of the WASH complex – to influence the proper dynamics of recruitment from the cytosol to the vesicle surface (Derivery et al., 2012).

In conclusion, we have found that Mroh1 is an actin-regulated protein that works with the WASH complex in late vesicular sorting. The conservation of its sequence and protein localization suggest that its role will be similar in other species; we look forward to finding out.

## MATERIALS AND METHODS

### Cell methods

Axenic *Dictyostelium* strains (a gift from Dr. Rob Kay, MRC-LMB, Cambridge, UK) were cultured in HL5 medium (Formedium). For imaging of vesicles, cells were incubated in HL5 medium containing 0.4 mg/ml TRITC–dextran (average molecular mass of 40 kDa; Sigma) with or without 0.2 mg/ml FITC–dextran (Sigma)+2% unlabelled dextran. For quantitative assays, cells were incubated in HL5 medium with 0.5 mg/ml TRITC–dextran overnight in sterile flasks shaking at 100 rpm. Cells were harvested by centrifugation (300 ***g*** for 3 min), washed once in fresh medium and resuspended in 12 ml fresh HL5. 0.9 ml samples were taken over a 6-h time course. Samples were centrifuged, cell pellets washed once in phosphate buffer, lysed in 0.1% TritonX-100, and samples were measured with a Photon Technology International fluorimeter using 544 nm excitation and 574 nm emission.

For FACS sorting, cells were labelled with 0.5 mg/ml TRITC–dextran overnight, then rinsed and the medium changed to fresh HL5. Cells were then incubated for 2–3 h to allow exocytosis of the TRITC–dextran. After this time, cells were harvested, washed once in 10 mM phosphate buffer pH 6.1, resuspended in phosphate buffer+10 mM EDTA, filtered through a 40 µm cell strainer to remove clumps, and then sorted on a BD FACSAria. Positive cells were collected and re-grown in HL5 until confluent. After a second round of sorting, cells were cloned using *K. aerogenes* on SM agar (Formedium) plates. Individual colonies were picked, re-grown in HL5 and tested for their exocytosis phenotype.

MEFs (a gift from Prof. Laura Machesky, The Beatson Institute, Glasgow, UK) were cultured in DMEM supplemented with L-glutamine at 37°C and 5% CO_2_ in a humid incubator. Cells were transfected with 5 μg plasmid DNA by using an Amaxa kit (Lonza).

### Molecular biology

A REMI library in Ax3 cells was generated using the plasmid pBSR2 cut with BamHI. DNA was co-transfected into cells with the DpnII restriction enzyme (King et al., 2010). Cells were dispensed into 96-well dishes in HL5 medium and Blasticidin (10 μg/ml) was added the next day. When clones appeared, they were harvested and stored as pools of 50 clones each. Frozen pools were revived into HL5 medium and expanded prior to use in the FACS selection protocol.

For gene identification of REMI mutants, genomic DNA was isolated (QiAamp DNA extraction kit, Qiagen) and digested with either AluI or NlaIII, then ligated in order to circularize fragments. Inverse PCR (Phusion, ThermoFisher Scientific) was performed using a number of outward-facing primers located within the pBSR2 plasmid sequence. Obtained PCR bands were cloned into either pDM368 or Blunt TOPO (Invitrogen, ThermoFisher Scientific) and sequenced.

Mroh1 gene disruption by homologous recombination used a KO construct encompassing nucleotides −756 to +2211 (5′ end) and +3188 to +5015 (3′ end) of the Mroh1 locus (numbering relative to ATG translation start site). A Blasticidin S resistance (BSR) cassette was cloned between these two flanking arms. Potential homologous recombinants were screened by PCR from genomic DNA, and two positives taken for further characterization. As the phenotype of the homologous recombinant clones was the same as the original REMI mutants, we used the recombinant clones for all subsequent experiments.

*Dictyostelium* genes of interest were identified at Dictybase (http://dictybase.org; Chisholm, 2006). DNA was amplified by PCR using PrimestarMax (TaKaRaClontech), cloned using Blunt TOPO and sequenced. Inserts were transferred to expression plasmids of interest and transfected into *Dictyostelium* cells by electroporation using a BTX Gene Pulser. Transfections were performed in 10 mM phosphate buffer pH 6.1 containing 50 mM sucrose, using a single pulse of 500 V. Selections were performed using 50 µg/ml Hygromycin B or 10 µg/ml G418 (Formedium) added the day after transfection.

The mouse Mroh1 cDNA was obtained from Source Bioscience (Table S2).

### Live-cell imaging

Cells were incubated overnight in glass bottom dishes (Mattek) in filtered HL5 medium containing 2% unlabelled dextran to swell vesicles. Confocal imaging was performed on one of four microscopes: a Zeiss LSM880 with an Airyscan detector, an Olympus FV1000, a Nikon A1R, or a Andor Spinning Disk coupled to a Nikon Eclipse microscope body. In all cases a 60× or 63×1.4 NA objective was used. Sequential imaging of different fluorescent channels was used where this was possible. Images were saved in the native format of the microscope.

For image analysis, files were opened in ImageJ (Schneider et al., 2012) or FiJi and saved as TIFFs. Adjustments to brightness were made as required. To assess localization of proteins on vesicles the Line tool and Plot Profile plugins were used. For measurement of proteins during exocytosis, we wrote an ImageJ plugin that positioned a band around the circumference of the vesicle, containing all the fluorescent signal (available from the corresponding author on request). The band was dynamically divided into 12 to 24 sectors, and the signal from each channel was measured. The results were plotted on a Radar Plot in Microsoft Excel, with each multiple of 12 or 24 sections representing the perimeter of the vesicle at any one time-point. For colocalization analysis, not done by these custom methods, CoLoc2 in Fiji was used.

FRAP recovery curves were analysed by fitting the data to a single-exponential model [Intensity(*t*)=A+B×exp(−C×*t*)], where *t* is the time from bleaching (sec) and Intensity(t) is the fluorescence intensity at time *t*. A, B and C were found by the method of nonlinear least squares. A Sharpiro–Wilk test showed that the recovery times were skewed; they were natural-log transformed and passed the Sharpiro–Wilk normality test (*P*=0.998 for Mroh1 and 0.093 for WASH). Geometric means and confidence intervals were calculated from the transformed data. Immobile fractions were normally distributed and calculated from the untransformed data.

### Immunoprecipitation and western blotting

GFP-Trap A resin (ChromoTek) was used to purify GFP fusion proteins from cells (25 µl of suspension per immunoprecipitation). For western blotting, we used anti-GFP (ab290; Abcam) at 1:1000, and a custom rabbit anti-Dd-WASH antibody (2898; Biogenes) at 1:200, with Protran 0.45 µm Nitrocellulose (GE Healthcare). Blocking was done in 5% fat-free milk in Tris-buffered saline (TBS). Primary antibodies were diluted in 5% bovine serum albumin (BSA) in TBS+0.1% Tween-20 (TBST). An anti-rabbit DyLight 800-conjugated secondary antibody (Thermo Scientific) was used at 1:10,000 in TBST. Membranes were scanned at 800 nm using a Li-Cor Odyssey CLx machine.
